# Diagnosis of Early Neurological Deterioration after Intravenous Thrombolysis for Patients with Cerebral Ischemic Stroke Using Compressed Sensing-Magnetic Resonance Imaging Algorithm

**DOI:** 10.1155/2021/2529936

**Published:** 2021-07-20

**Authors:** Junjun Wang, Benxiao Wang, Yongliang Tang, Hui Yan

**Affiliations:** Department of Neurology, General Hospital of Wanbei Coal and Electricity Group, Suzhou 234000, Anhui, China

## Abstract

This study was to explore the risk factors and prognosis of early neurological deterioration (END) after intravenous thrombolysis in patients with cerebral ischemic stroke (CIS) with the guidance of magnetic resonance imaging (MRI) under the compressed sensing-MRI (CSMRI) algorithm. 187 patients with CIS in the hospital were selected and grouped into a deterioration group and a control group according to whether they had END. The CSMRI algorithm was constructed and compared with digital television (DTV) algorithm and Bayesian compressed sensing (BCS) algorithm. It was found that the reconstruction time of CSMRI algorithm in platform I (1134.9 s) and platform II (2615.8 s) was visibly lower than that of DTV algorithm (2634.6 s, 3963.4 s) and BCS algorithm (5631.5 s, 7412.3 s), showing statistically obvious differences (*P* < 0.05). In addition, the reconstruction efficiency of the CSMRI algorithm was the best. After 4 hours of intravenous thrombolysis, the stroke scale score (12.3 scores) of the deterioration group was much higher than that of the control group (8.4 scores) (*P* < 0.05). The occlusion of responsible great vessel in the deterioration group (30 cases, 83.33%) was obviously higher in contrast to that in the control group (74 cases, 49%) (*P* < 0.05). Stroke scale score and occlusion of responsible great vessel were risk factors for EBD after intravenous thrombolysis.

## 1. Introduction

Cerebral ischemic stroke (CIS), also known as cerebral infarction, is one of the most common types of stroke in clinical practice [1]. The global mortality rate of stroke is second only to malignant tumors, and it is the disease with the highest mortality rate in China. CIS accounts for about 75% of strokes all over the country, and its death toll accounts for about 15% of all deaths. Even if 70% of stroke patients survive, they will also cause varying degrees of disability [2]. Nowadays, the incidence of CIS is still rising, with approximately 1.1 million new CIS patients every year, which seriously threatens the lives and health of patients and brings a heavy financial burden to families [3].

At present, intravenous thrombolysis is recognized as the best way to treat CIS [4]. Li et al. [5] used alteplase intravenous thrombolysis treatment within 3 hours of the onset of CIS, which had a good clinical effect. However, its efficacy is affected by many factors. Some patients still experience the early neurological deterioration (END) after thrombolytic therapy, which not only prevents patients from benefiting from it but also leads to increased neurological damage. Therefore, scholars are required to explore its risk factors and adjust the time of drug treatment and the treatment dose.

With the continuous advancement of medical imaging technology in recent years, the early diagnosis of CIS has been greatly improved. Among them, magnetic resonance imaging (MRI) can clarify the patient's onset time and assess whether the patient is suitable for intravenous thrombolysis treatment, thereby reducing the occurrence of comorbidities [6]. Compressed sensing is a “collect-compress-build” theory, which can perform high-efficiency signal reconstruction on a small number of sets according to the sparse characteristics of the signal [7]. The compressed sensing framework is applied to MRI to form the compressed sensing-MRI (CSMRI). This has been an emerging technology in the recent years. As Chen et al. [8] discussed in their article in 2020, the deep learning model is increasingly widely used in our lives. Using the sparse signal sensing function and reconstruction mechanism of the compressed sensing theory, the amount of data can be reduced, thereby improving the efficiency of magnetic resonance imaging [9]. Compared with other methods, CSMRI does not require additional high costs, reduces the psychological burden of patients during the examination, and adopts real imaging to the greatest extent, with good image reconstruction effects [10].

In this study, the CSMRI algorithm was constructed and compared with the digital television (DTV) algorithm and BCS algorithm and applied to MRI images of 187 CIS patients. The objective of this study was to explore the risk factors that affect END after thrombolytic therapy in CIS patients. Controlling the risk factors can realize the early treatment and diagnosis of neurological deterioration, and the prognosis of patients can be improved. This has a very important reference for clinical treatment and prevention of END.

## 2. Materials and Methods

### 2.1. Research Objects

245 patients with CIS who were diagnosed in hospital from October 15, 2017, to December 10, 2020, were selected as the research subjects, and intravenous thrombolysis treatment was performed within 3 hours of onset. Patients with END were included in the deterioration group, and those with no END were regarded as the control group. The study was approved by the Medical Ethics Committee of the hospital, and the patients and their families had understood the research situation and signed the informed consent forms.

The inclusion criteria were defined as follows: patients whose diagnosis met the diagnostic criteria of CIS, causing neurological damage; patients whose duration of stroke symptoms was not less than 30 minutes; patients whose computed tomography (CT) examination showed no intracranial hemorrhage; and patients who received intravenous thrombolysis within 3 hours of onset.

The exclusion criteria were defined as follows: patients with neurological impairment caused by epileptic seizures; patients with major diseases such as heart, liver, and kidney; patients with major surgery one month before and after the experiment; and patients with internal tumors and aneurysms.

### 2.2. Collection of Research Information

  Clinical data: age, gender, blood pressure, past disease history (hypertension, diabetes, coronary heart disease, cardiac insufficiency, smoking, and drinking), and *National Institutes of Health Stroke Scale* (NIHSS) score (4 hours before thrombolysis, immediately after thrombolysis, and 4 hours, 24 hours, and 1 week after thrombolysis).
  Laboratory indicators: white blood cell count (WBC) (×10^9^/L), platelet count (PLT) (×10^9^/L), emergency blood glucose (mmol/L), international normalized ratio (INR), low-density lipoprotein (g/L), high-density lipoprotein (g/L), hemoglobin (g/L), and homocysteine (mol/L).
  Imaging data: MRI early cerebral infarction, responsible infarct location (anterior circulation stroke and posterior circulation stroke), distribution of responsible great arteries (internal carotid artery and middle cerebral artery), and occlusion degree of responsible great vessel (normal, stenosis, and occlusion).

### 2.3. Experimental Evaluation Method

The diagnosis of CIS referred to the diagnostic criteria in the *China CIS Diagnosis and Treatment Guidelines* 2014. Neurological impairment was assessed using the NHISS. Patients were given baseline NIHSS scores immediately after admission, immediately after thrombolysis, and 3 hours, 24 hours, and 1 week after thrombolysis. The NIHSS score was widely used in evaluating CIS patients. The reliability of each individual item in the NIHSS score was better, but the overall total score was quite different. In order to avoid the change of NIHSS score affecting the results, the ΔNIHSS ≥4 scores were adopted for the occurrence of general END. The baseline NIHSS score was a diagnostic criterion for assessing the occurrence of END. Different baseline NIHSS scores and different diagnostic criteria were adopted for evaluation of END so as to improve the accuracy of diagnosis. If the baseline NIHSS score was less than 6 scores, it was recorded as increase of 2 or above scores within 24 hours after intravenous thrombolysis. If the baseline NIHSS score was ≥6 scores, it was recorded as increase of 4 or above scores within 24 hours after intravenous thrombolysis.

### 2.4. CSMRI Evaluation

The MR Prisma 3.0 magnetic resonance instrument produced by Siemens (Germany) was used to evaluate patients. When scanning, the patient was required to keep a supine position, maintain steady breathing, tilt the head slightly back, and fully expose the neck. The patient was scanned with the linear array probe from the proximal end to the distal end and the examination procedure was introduced to them. The scanning parameters included the matrix of 251 × 251, the layer thickness of 3.5 mm, the field of view of 25 × 25 cm, the flip angle of 15°, and the layer spacing of 6.1 mm.

### 2.5. CSMRI Algorithm

Compressive sensing theory tried to design a measurement matrix Π. The vector *B* to be measured can be obtained through linear measurement and *N* operations. Among them, the measurement matrix Π existed alone, and the specific measurement equation is expressed as follows:

(1) $$\begin{array}{c} B=\Pi A=\Pi \Omega T=\Theta T. \end{array}$$

In the above equation, Π represents the measurement matrix, *B* represents the measurement vector, *A* represents the sparse signal, Θ=ΠΩ refers to the matrix of *M* × *N*, and Ω refers to the sparse mapping matrix. Compressed sensing theory can use equation (1) to reconstruct sparse signal *A*. According to the measurement process and matrix calculation method, rank(Θ) < *M*, and the infinite solution of equation (1) can be obtained, which was defined as *a*_0_; then equation (2) could be obtained:

(2) $$\begin{array}{c} a^{\prime }=a_{0}+a^{*},a^{*}\in M,M=\left\{a|\Theta a=0\right\}. \end{array}$$

In the above equation, *M* represented the null space of the measurement matrix Π. Column(Θ) was defined as the smallest number of linearly correlated columns with matrix Θ; then it could satisfy Column(Θ) > 2*k*. There was a unique *k*-sparseness for equation (1); that is, the number of nonzero vectors of vector *A* was lower than *k*. *M*∩*Q*_2*k*_={0} could be obtained based on Column(Θ) > 2*k*. *Q*_*k*_ referred to a space for the combination degree of multiple linear subspaces. It was assumed that there were two *k*-sparse solutions of the linear equation *B*=Θ*A*, and the following equation is satisfied:

(3) $$\begin{array}{c} \Theta a_{1}=\Theta a_{2}=B \end{array}$$

In equation (3) above, *a*_1_ and *a*_2_ represent two different *k*-sparse solutions, both of which belong to *Q*_*k*_; then (*a*_1_ − *a*_2_) was included in *Q*_2*k*_. The signal framework of compressed sensing theory was essentially to optimize the constructed model based on the detection data and sparse information, and then obtain the best solution. The common framework of sparse signal based on CSMRI could be expressed as follows:

(4) $$\begin{array}{c} \underset{A}{\min}\sum_{i}\eta_{i}\phi_{i}\left(A\right)t.s.{\left\|\text{EA}-C\right\|}_{2}^{2}\leq \lambda . \end{array}$$

In the equation above, *A* represents the sparse signal to be recovered, *E* represents downsampling and Fourier mapping, *λ* refers to the noise pollution level, *ϕ*_*i*_(·) is the sparse constraint term, and *η*_*i*_ is used to balance the data fidelity term. The Lagrangian form could be expressed as follows:

(5) $$\begin{array}{c} \underset{A}{\min}\left\{\frac{1}{2}{\left\|\text{EA}-C\right\|}_{2}^{2}+\sum_{i}\eta_{i}\phi_{i}\left(A\right)\right\}. \end{array}$$

In equation (5) above, *A* represents the sparse signal to be recovered, *E* represents downsampling and Fourier mapping, *η*_*i*_ is used to balance the data fidelity term, and *ϕ*_*i*_(·) represents the sparse constraint term. In CSMRI, there were many forms for *ϕ*_*i*_(·), indicating that the sparseness of multiple structures was one of the characteristics of MRI.

### 2.6. Design of the Simulation Experiment

In this experiment, the *L*_1_ and *L*_2_ norm minimization is used to construct the image due to the sparse nature of the image in the total variational mapping space. The constructed model is as follows:

(6) $$\begin{array}{c} \underset{A}{\min}\left\{{\left\|\text{SW}\left(A\right)\right\|}_{1}\right\}t.s.\text{EA}=B,\\ \underset{A}{\min}\left\{{\left\|\text{SW}\left(A\right)\right\|}_{2}\right\}t.s.\text{EA}=B. \end{array}$$

*E* represents the measurement matrix, taking Fourier sparseness as the sampling template, and SW(·) represents Fourier downsampling. Based on the actual experimental results, the reconstruction effect of minimization of the norm *L*_1_ was better than that of *L*_2_.

### 2.7. Statistical Methods

SPSS 22.0 statistical software was used to calculate and analyze the data. The calculated data conforming to the normal distribution were represented by the mean ± standard deviation ($\bar{x}\pm s$), and other calculated data were represented by the percentage (%). The baseline data comparison between the two groups was analyzed with the single factor analysis, and the measurement data conforming to the normal distribution were compared with *t*-test of two independent samples. In the univariate analysis, the multivariate logistic regression analysis model was adopted when *P* < 0.2 variables. Multivariate regression analysis was employed to explore the risk factors that affect END. *P* < 0.05 meant the statistical difference could be found.

## 3. Results

### 3.1. Comparison on Reconstruction Time of Three Algorithms

**Figure 1:**
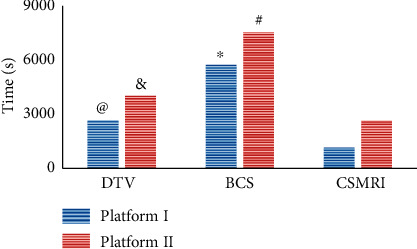
Comparison of reconstruction times of three algorithms. *Note.*^*∗*^and ^#^ suggest that the difference was observable compared with DTV algorithm in platform I and platform II, respectively (*P* < 0.05); @ and & indicate that the visible difference could be found in contrast to the BCS algorithm, respectively (*P* < 0.05).

The CSMRI algorithm was constructed and compared with the DTV algorithm and BCS algorithm and then applied to MRI images of 187 CIS patients. The results showed that the reconstruction times of DTV algorithm in platform I and platform II (2634.6 s, 3963.4 s) were much lower than those of BCS (5631.5 s, 7412.3 s) (*P* < 0.05) (Figure 1). The reconstruction times of CSMRI algorithm in platform I (1134.9 s) and platform II (2615.8 s) were visibly lower than those of DTV algorithm (2634.6 s, 3963.4 s) and BCS algorithm (5631.5 s, 7412.3 s) (*P* < 0.05). It was shown that the reconstruction efficiency of the DTV algorithm was higher than that of the BCS algorithm; the reconstruction efficiency of the CSMRI algorithm was the highest, so that it could make full use of the information in the time and space domain, showing a good reconstruction effect.

### 3.2. Comparison of Baseline Data of Patients

**Figure 2:**
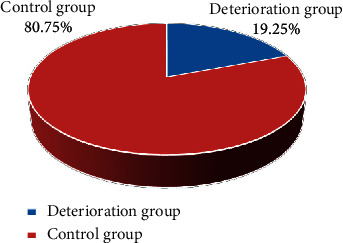
Proportion of patients in two groups.

**Figure 3:**
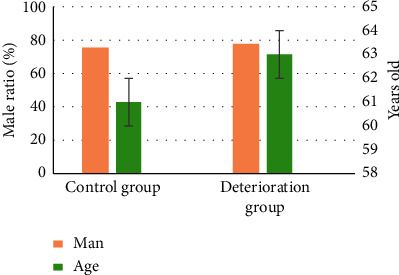
Comparison of proportion of males and age of patients.

245 patients were treated with alteplase intravenous thrombolysis within 3 hours of onset, and 187 patients were finally enrolled. Among them, 36 patients had END, with an incidence of 18.36%, and they were included in the deterioration group; 151 patients (77.04%) who had no END were set as the control group (as shown in Figure 2). The deterioration group included 28 males (77.78%) and 8 females (22.22%), with an average age of 63 ± 14 years; the control group had 114 males (75.5%) and 37 females (24.5%), with an average age of 61 ± 12 years. Thus, no observable difference could be found between the two groups of patients (*P* > 0.05) (as illustrated in Figure 3).

### 3.3. Single Factor Analysis on END

**Figure 4:**
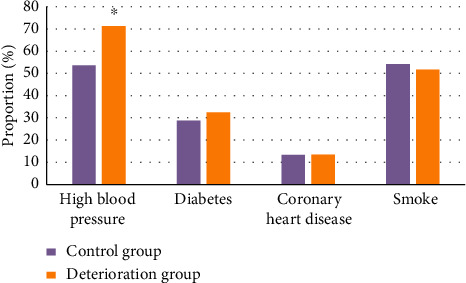
Comparison of clinical manifestations of patients. *Note.*^*∗*^suggests that the difference was huge in contrast to the control group (*P* < 0.05).

**Figure 5:**
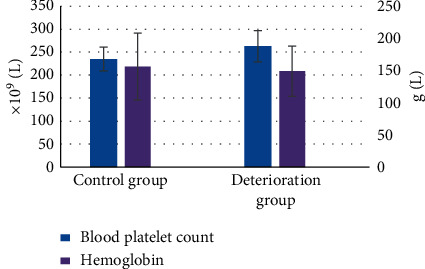
Comparison of PLT and hemoglobin level in patients. *Note.*^*∗*^suggests that the difference was huge in contrast to the control group (*P* < 0.05).

The baseline data and laboratory indicators of patients in the deterioration group and the control group revealed that there was no great difference in diabetes, coronary heart disease, and smoking between the two groups (*P* > 0.05). The ratio of hypertension in the deterioration group (71.2%) was greatly higher in contrast to that in the control group (53.6%) (*P* < 0.05) (Figure 4). The WBC count, emergency blood glucose, INR, low-density lipoprotein, high-density lipoprotein, hemoglobin, and homocysteine levels were not different greatly in patients between two groups (*P* > 0.05). The PLT count of the deterioration group (263 ± 39 g/L) was much higher in contrast to that in the control group (235 ± 52 g/L) (*P* < 0.05) (as shown in Figure 5). It indicated that patients with hypertension were more likely to have END, and the number of platelets could be undertaken as a predictor of END.

### 3.4. MRI Image of CIS

**Figure 6:**
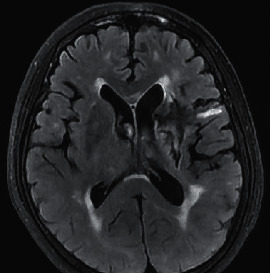
MRI image of a 65-year-old male patient in acute stage (cerebrovascular hyperdensity and disappeared invisible sulci).

**Figure 7:**
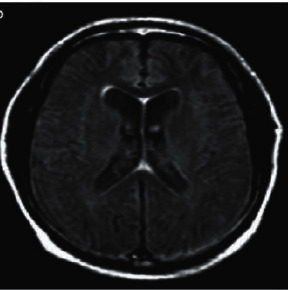
MRI image of a female patient (60 years old) in chronic stage (decreased brain volume and gliosis).

The imaging characteristics of different stages of CIS could be described as follows. In the acute stage (within 24 hours), the initial CT findings were mild, including hyperdensity of blood vessels, unclear boundaries of gray matter, and invisible sulci (as shown in Figure 6). The diffusion-weighted imaging was highly accurate and could detect stroke 15 minutes after the onset. T2/fluid attenuated inversion recovery (T2/FLAIR) high signal began to appear a few hours later. In the subacute phase (24 hours to 2 months), false normal images could be obtained on the 4^th^∼10^th^ day. The gyri-like enhancement could be found on the 6^th^ day and lasted 2 to 3 months. The peak of edema appeared in the 3^rd^∼4^th^ day and began to decrease after the 7^th^ day. Hemorrhagic transformation occurred at the 2^nd^∼7^th^ day after the onset. The chronic phase (after 2 months) was characterized by brain volume reduction, cavitation, and gliosis (as shown in Figure 7). Glial hyperplasia surrounding cavities showed low density on CT and showed high signal on T2-weighted imaging (T2WI) and FLAIR.

### 3.5. Treatment Effect of Alteplas Intravenous Thrombolysis in Patients in Two Groups

**Figure 8:**
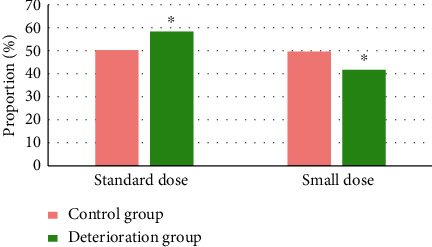
Comparison of alteplase dose in different groups. *Note.*^*∗*^suggests that the difference was huge in contrast to the control group (*P* < 0.05).

The standard dose of alteplase was 0.9 mg/mL, the maximum dose was 80 mg, the small dose was 0.5∼0.8 mg/mL, and the maximum dose was 45 mg (Figure 8). The proportion of patients in the deterioration group who used the standard dose (58.3%) was observably higher in contrast to 50.3% in the control group (*P* < 0.05); the proportion of patients in the deterioration group who used the low dose (41.7%) was in a lower level in contrast to the control group (*P* < 0.05). It indicated that the dose of alteplase was a potential risk factor affecting the END in patients with CIS.

### 3.6. Comparison of NIHSS Scores

**Figure 9:**
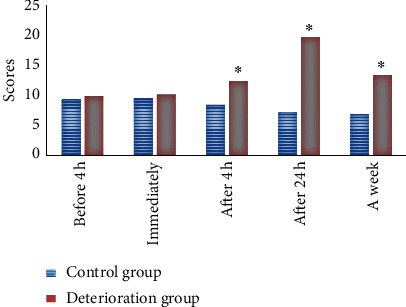
NIHSS scores of patients at different times. *Note.*^*∗*^suggests that the difference was huge in contrast to the control group (*P* < 0.05).

There was no remarkable difference in NIHSS scores between the two groups of patients 4 hours before and immediately after alteplase thrombolysis treatment (*P* > 0.05). After intravenous thrombolysis treatment for 4 hours, the NIHSS score in deterioration group (12.3 scores) was higher than the score in the control group (8.4 scores) (*P* < 0.05). After intravenous thrombolysis treatment for 24 hours and one week, the NIHSS scores in the deterioration group were 19.6 and 13.3, respectively, which were obviously higher in contrast to scores of 7.2 and 6.8 in the control group (*P* < 0.05) (as illustrated in Figure 9).

### 3.7. Statistical Comparison of Image Data of Patients

**Figure 10:**
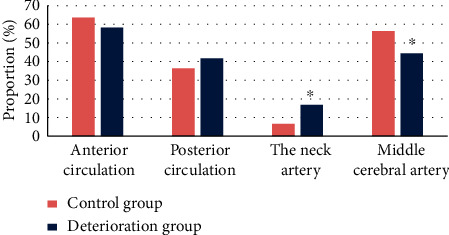
Comparison of responsible infarct sites and distribution of great arteries of patients. *Note.*^*∗*^suggests that the difference was huge in contrast to the control group (*P* < 0.05).

In the deterioration group, 21 patients (58.3%) had anterior circulation stroke at the responsible infarct site and 15 patients (41.7%) had posterior circulation stroke; 96 patients (63.6%) had anterior circulation stroke and 55 patients (36.4%) had posterior circulation stroke in the control group. Thus, no visible difference could be found between the two groups (*P* > 0.05). In the deterioration group, 6 cases (16.7%) showed the responsible aorta in the internal carotid artery, while there were 10 cases (6.62%) in the control group, showing notable difference (*P* < 0.05); 16 cases (44.4%) showed responsible aorta in artery in the deterioration group, while there were 85 cases (56.3%) in the control group, showing remarkable difference (*P* < 0.05) (as revealed in Figure 10).

**Figure 11:**
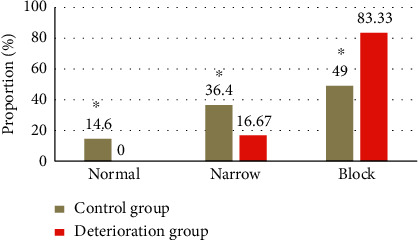
Comparison of the occlusion degree of responsible great vessel of patients. *Note.*^*∗*^suggests that the difference was huge in contrast to the control group (*P* < 0.05).

There were 30 cases (83.33%) that suffered from occlusion of responsible great vessel in the deterioration group, which was much higher in contrast to the control group (74 cases, 49%), showing statistical difference (*P* < 0.05); there were 6 cases (16.67%) that suffered from narrow responsible great vessel in the deterioration group, which was much lower in contrast to the control group (55 cases, 36.4%), showing observable difference (*P* < 0.05) (as disclosed in Figure 11).

### 3.8. Multivariate Logistic Regression Analysis on Risk Factors of END

In univariate analysis, variables with *P* < 0.2 were undertaken as independent variables, and a two-class logistic regression analysis was adopted for multivariate regression analysis of the NIHSS score and occlusion of responsible great vessel (Table 1). The results showed that each increased 1 point in the NIHSS score meant OR = 1.301, 95% confidential interval (CI) = 1.023∼1.254, and *P*=0.03; for occlusion of responsible great vessel, OR = 12.419, 95% CI = 2.361∼43.328, and *P*=0.002. NIHSS score and occlusion of responsible great vessel were correlated with the occurrence of END (*P* < 0.05).

## 4. Discussion

The pathogenesis of CIS lies in cerebral infarction caused by occlusion of cerebral vasculature. In recent years, foreign clinical controlled experiments have confirmed that CIS is highly effective with intravenous thrombolysis treatment within 3 hours of onset of CIS [11].

Khazaei et al. [12] investigated the risk factors of END after intravenous thrombolysis treatment and found that the average NIHSS score of the 234 patients enrolled increased by more than 4 points within 24 hours after the onset of the disease, of which 36 patients suffered from END, so the incidence of END was 15.38%. Simonsen et al. [13] explored the relationship between END and NIHSS score 24 hours after the end of alteplase thrombolytic therapy and defined patients whose NIHSS score increased by more than 4 points as suffering from END; and the incidence rate of END was 36.1%. END in this study was defined as an increase in the NIHSS score by more than 4 points. Among the 196 patients enrolled, 36 patients suffered from END, with an incidence rate of 18.36%, which was basically consistent with the above results.

The results here showed that the NIHSS score was a risk factor for END after intravenous thrombolysis treatment, so the risk of END will rise with the increase of NIHSS score. Irvine et al. [14] included 865 patients who were treated with alteplase intravenous thrombolysis for the treatment of CIS and found that the higher the NIHSS score 24 hours and 72 hours after the end of thrombolysis, the more severe the nerve damage in stroke patients and the worse the state. However, Fujita et al. [15] explored the influencing factors of END after intravenous thrombolysis treatment with alteplase and found that the lower the NIHSS score, the higher the risk of END. Prochazka et al. [16] included 364 patients with CIS who underwent intravenous thrombolysis within 3 hours of onset to explore the incidence, risk factors, and prognosis of END, and the results showed that low-level NIHSS scores indicated the incidence of END. The different results of the above studies may be related to the upper limit of the scale. A high level of NIHSS score indicates that the patient's neurological impairment is severe, and the degree of deterioration is high. A low level of NIHSS score indicates that the degree of deterioration of the patient will increase continually. Therefore, CIS patients should pay attention to their physical signs after intravenous thrombolysis treatment, even if the NIHSS score is low.

## 5. Conclusion

The CSMRI algorithm was constructed and compared with the DTV algorithm and BCS algorithm and then applied to MRI images of 187 CIS patients. The reconstruction efficiency of CSMRI algorithm was higher in contrast to the DTV algorithm and BCS algorithm. The incidence of END was 18.36%. NIHSS score was a risk factor for END after intravenous thrombolysis treatment, and the risk of END increased with the elevation of NIHSS score. Occlusion of responsible great vessel was another risk factor for END after intravenous thrombolysis. However, the patients with severe ischemic cerebral anemia and stroke cannot be excluded for analysis of the impact data, which affected the results of the study. Later, the condition of the included patients will be improved for further exploration. In conclusion, the risk factors of END after intravenous thrombolytic therapy in patients with hemorrhagic stroke were determined in this study. Based on the control of risk factors, early treatment and diagnosis of early neurological deterioration can be achieved, and the prognosis of patients can be improved. This study showed very important reference significance for clinical treatment and prevention of early deterioration of neurological function.

## Figures and Tables

**Table 1:** Results of multivariate regression analysis.

	NIHSS score	Occlusion of responsible great vessel
B value	0.214	2.364
Wald value	9.451	9.364
OR value	1.301	12.419
95% CI	1.023∼1.254	2.361∼43.328
*P* value	0.03^*∗*^	0.002^*∗*^

*Note.* ^*∗*^indicates that it was correlated with the occurrence of END.

## Data Availability

No data were used to support this study.
